# Extracorporeal Life Support and Temporary CentriMag Ventricular Assist Device to Salvage Cardiogenic-Shock Patients Suffering from Prolonged Cardiopulmonary Resuscitation

**DOI:** 10.3390/jcm11133773

**Published:** 2022-06-29

**Authors:** Jia-Lin Chen, Yi-Ting Tsai, Chih-Yuan Lin, Hong-Yan Ke, Yi-Chang Lin, Hsiang-Yu Yang, Chien-Ting Liu, Shih-Ying Sung, Jui-Tsung Chang, Ying-Hsiang Wang, Tso-Chou Lin, Chien-Sung Tsai, Po-Shun Hsu

**Affiliations:** 1 Department of Anesthesia, Tri-Service General Hospital, National Defense Medical Center, Taipei 11490, Taiwan; babe.ane@gmail.com (J.-L.C.); tclin@mail.ndmctsgh.edu.tw (T.-C.L.); 2 Division of Cardiovascular Surgery, Department of Surgery, Tri-Service General Hospital, National Defense Medical Center, Taipei 11490, Taiwan; cvsallen@mail.ndmctsgh.edu.tw (Y.-T.T.); linrock@ms26.hinet.net (C.-Y.L.); drkehy@yahoo.com.tw (H.-Y.K.); m860630@mail.ndmctsgh.edu.tw (Y.-C.L.); alfie0314@gmail.com (H.-Y.Y.); justin.liu@yahoo.com.tw (C.-T.L.); molecule1983@gmail.com (S.-Y.S.); rayzone7474@gmail.com (J.-T.C.); imma031@yahoo.com.tw (Y.-H.W.)

**Keywords:** CentriMag, heart failure, cardiogenic shock, extracorporeal life support, extracorporeal membrane oxygenation, extracorporeal cardiopulmonary resuscitation, ventricular assist device

## Abstract

**Background**: The extracorporeal life support (ECLS) and temporary bilateral ventricular assist device (t-BiVAD) are commonly applied in patients with cardiogenic shock. Prolonged cardiopulmonary resuscitation (CPR) has poor prognosis. Herein, we report our findings on a combined ECLS and t-BiVAD approach to salvage cardiogenic-shock patients with CPR for more than one hour. **Methods**: Fifty-nine patients with prolonged CPR and rescued by ECLS and subsequent t-BiVAD were retrospectively collected between January 2015 and December 2019. Primary diagnoses included ischemic, dilated cardiomyopathy, acute myocardial infarction, post-cardiotomy syndrome, and fulminant myocarditis. The mean LVEF was 16.9% ± 6.56% before t-BiVAD. The median ECLS-to-VAD interval is 26 h. **Results**: A total of 26 patients (44%) survived to weaning, including 13 (22%) bridged to recovery, and 13 (22%) bridged to transplantation. Survivors to discharge demonstrated better systemic perfusion and hemodynamics than non-survivors. The CentriMag-related complications included bleeding (*n* = 22, 37.2%), thromboembolism (*n* = 5, 8.4%), and infection (*n* = 4, 6.7%). The risk factors of mortality included Glasgow Coma Scale (Motor + Eye) ≤ 5, and lactate ≥ 8 mmol/L at POD-1, persistent ventricular rhythm or asystole, and total bilirubin ≥ 6 mg/dL at POD-3. Mortality factors included septic shock (*n* = 11, 18.6%), central failure (*n* = 10, 16.9%), and multiple organ failure (*n* = 12, 20.3%). **Conclusions**: Combined ECLS and t-BiVAD could be a salvage treatment for patients with severe cardiogenic shock, especially for those already having prolonged CPR. This combination can correct organ malperfusion and allow sufficient time to bridge patients to recovery and heart transplantation, especially in Asia, where donation rates are low, as well as intracorporeal VAD or total artificial heart being seldom available.

## 1. Introduction

Cardiogenic shock is a low-cardiac-output state, which always leads to systemic end-organ hypoperfusion and acute pulmonary edema in the presence of severe left ventricular dyskinesia. It usually results in life-threatening complications that induce a high mortality rate [1]. Prognosis is poor with cardiopulmonary resuscitation (CPR), especially when the patient has suffered from prolonged CPR [2]. Rajan et al. reported that the 30-day survival for CPR duration of more than 25 min was only 13.8%. Currently, various options of temporary mechanical circulatory support (MCS) exist for the treatment of cardiogenic shock. However, none of these contemporary MCS options offer convincing evidence that it could improve survival rate [3]. Extracorporeal life support (ECLS) is the most common temporary MCS applied in the emergency setting. It serves as a closed-system heart–lung machine, also called cardiopulmonary bypass, which can immediately provide temporary systemic oxygenation and circulatory support. However, ECLS does have a limited flow, does increase the cardiac afterload, and cannot unload the left ventricular tension, which may worsen pulmonary circulation and coronary perfusion [4]. Therefore, temporary bilateral ventricular assist devices (t-BiVAD) should be applied to replace ECLS in this “crash and burn” population [5,6]. A CentriMag heart pump (Thoratec Corporation) has been used as a temporary VAD since 2006 [7], which can generate 10 L/min continuous flow and provide 75–100% cardiac support. Moreover, it can provide versatile mechanical support for right, left, or bi-ventricular failure, as well as pulmonary support when combined with a membrane oxygenator [3]. However, concrete data on the application of CentriMag as short-term VAD for acute cardiogenic shock remain scarce, especially in Asian populations. Therefore, the aim of this study was to evaluate the outcomes, survival, and risk factors of mortality of the combined ECLS and t-BiVAD approach to salvage cardiogenic-shock patients who had suffered from CPR for more than one hour. 

## 2. Methods 

### 2.1. Patient Enrollment Criteria

**Figure 1:**
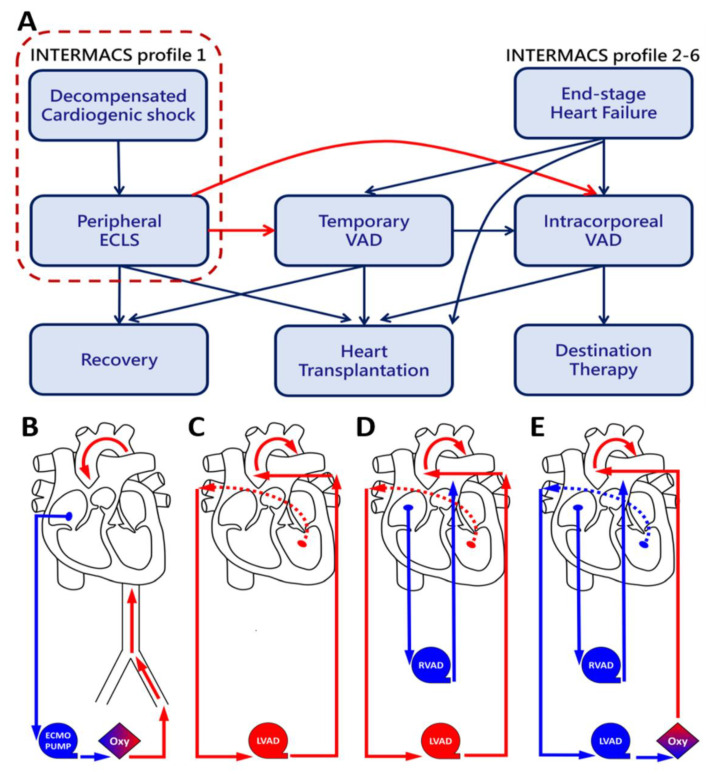
Treatment algorithm and device configurations. Each panel illustrates the blood flow direction of different configurations. (**A**) Treatment algorithm for acute cardiogenic shock and decompensated chronic heart failure according to different INTERMACS score and various clinical scenario. (**B**) Peripheral ECLS. ECLS Inflow: the deoxygenated blood (blue) comes from right atrium. ECLS Outflow: the oxygenated blood (red) flows into femoral artery. (**C**) Single LVAD. LVAD Inflow: the oxygenated blood (red) comes from left atrium or ventricle. LVAD Outflow: the oxygenated blood (red) flows into ascending aorta. (**D**) BiVAD without oxygenator spliced. Adding RVAD in configuration-C. RVAD Inflow: the deoxygenated blood (blue) comes from right atrium. RVAD Outflow: the deoxygenated blood (blue) flows into pulmonary artery. (**E**) BiVAD with oxygenator spliced. Adding oxygenator in configuration-D. ECLS, extracorporeal life support; INTERMACS, Interagency Registry for Mechanically Assisted Circulatory Support; LVAD, left ventricular assist device; RVAD, right ventricular assist device.

We retrospectively reviewed our experience of in-hospital cardiac arrest patients, who had witnessed prolonged CPR for more than 60 min without ROSC between January 2015 and December 2019. The data were collected by means of reviewing medical records during hospitalization and follow-up after discharge. This study was approved by our Institutional Review Board of Tri-Service General Hospital, National Defense Medical Center (TSGHIRB No. A202005092, Date of Approval: 5 June 2020) and all methods were performed in accordance with the relevant guidelines and regulations. Informed consent was waived by the ethics committee based on retrospective review of medical records. According to our treatment strategy of chronic heart failure and ECPR for decompensated cardiogenic shock (Figure 1A–D), CPR would stop once emergent ECLS was implemented. Unconscious patients were routinely treated with hypothermia 32–34 °C for 12–24 h using a heater–cooler device on ECLS, and brain computerized tomography (CT) was used to assess neurologic deficits. In the absence of brain tissue swelling on CT scan, subsequent t-BiVAD implementation (CentriMag, Levitronix LLC, Waltham, MA, USA) would be routinely considered in cases with low ECLS flow, LV distension with frequent or sustained VT, large dosage of inotropes, persistent pulmonary congestion, and systemic malperfusion despite maximal ECLS support, such as exacerbated renal and hepatic function. We routinely performed median sternotomy for CentriMag implantation. Both inflow and outflow cannulas were inserted with double-layer purse-string 3-0 Prolene sutures with pledgets and fixed with double snares for additional stability. A Medtronic EOPA 77722, 22 Fr outflow cannula was used for the aorta and the pulmonary artery, while a Medtronic DLPTM 68132, 32 Fr inflow cannula was used for the right superior pulmonary vein and right atrium. The configuration is shown in Figure 1E. Usually, severe pulmonary edema develops in critical cardiogenic shock, especially in patients with CPR and ECLS. The oxygenator could be spliced on either the RAVD or LAVD route. In our experience, the oxygenator spliced on LVAD could achieve optimal oxygenation of visceral organs. Once the pulmonary edema subsided and the oxygen debt during cardiogenic shock had been repaid, the oxygenator could be easily removed in the ICU.

### 2.2. Postoperative Care and Anticoagulation

The Glasgow Coma Scale (GCS) was used to assess the conscious level of patients on a daily basis. Due to endotracheal intubation, we only recorded eye opening and motor response without verbal response, which was excluded in GCS scoring. The LVAD flow was kept at 5.0–5.5 lpm with 3300–3500 rpm, while RVAD was kept at 0.5–1.0, lpm lower than LVAD, to prevent the development of pulmonary edema. The mean arterial pressure, central vein pressure, and pulmonary capillary wedge pressure (PCWP) were maintained at 75–90 mmHg, 12–15 mmHg and 12–15 mmHg, respectively. The oxygenator could be easily disassembled in the ICU as long as pulmonary edema was resolved. Usually, we kept inotropes at 5 mcg/kg/min to maintain LV outflow and prevent blood stasis and subsequent thrombus formation over LV and aortic root. During the VAD period, the ventilator was set at 40% FiO_2_ with PEEP 8 cmH_2_O to avoid alveolar collapse. The support pressure was set to 12–15 cmH_2_O so as to ensure an optimal tidal volume status (6–8 mL/kg), and the plateau pressure was controlled under 24 cmH_2_O to prevent barotrauma. Extubation was also considered in accordance with the American Thoracic Society guidelines. Systemic heparinization was performed to maintain active clotting time (ACT) around 160–180 s. Routine broad-spectrum prophylactic antibiotic was also used to prevent cannula-related infection, and the end-organ perfusion and function was monitored. Enteral or parenteral nutrition was also started. Once neurologic function was preserved, the CentriMag VAD functioned as a bridge to recovery, heart transplantation, or permanent intracorporeal VAD (Figure 1A). In cases of neurologic damage during cardiogenic shock, hospice with CentriMag withdraw was suggested when cardiac recovery was impossible. Central failure was diagnosed via repeated brain CT scans, as well as defined by a complete clinical neurologic examination, including coma documentation, absence of brain-stem reflexes, and apnea. The decision-making processes were also based on renal, hepatic, and respiratory recovery and on the absence of any infections. Daily bedside echocardiography was performed to assess cardiac function. Meanwhile, daily laboratory data were examined to assess systemic oxygenation, inflammation, and perfusion of each organ, especially on postoperative day 1 (POD-1) and day 3 (POD-3), which were recorded, analyzed, and compared.

### 2.3. Device Weaning and Removal

Weaning of CentriMag VAD was only considered in cases where the inotropes were tapered and systemic perfusion was adequate. ACT was adjusted up to more than 200 s via systemic heparinization before lowering the CentriMag revolution. For RVAD, weaning was always initiated prior to LVAD. RVAD weaning was discontinued in cases where signs of RV overload, such as RV distension and left deviation of septum by echocardiography, and frequent suction event concomitant with decreased flow of LVAD, were noted. Once RVAD weaning was successful, LVAD weaning was then initiated. For LVAD, weaning was considered in cases of the LVEF > 40%. The flow was reduced gradually to 1.5 L/min with a rate of 200 mL per day. LVAD weaning was discontinued if signs of LV overload, such as pulmonary edema by chest film, LV distension by echocardiography, and high PCWP by Swan-Ganz catheter (usually > 18 mmHg), were noted. Withdrawal and removal of Bi-VAD occurred when both RVAD and LVAD were weaned off. Otherwise, patients were referred for transplantation or durable intracorporeal VAD.

## 3. Statistical Analysis

SPSS 25.0 statistical software (SPSS Inc., Chicago, IL, USA) was used for all analyses, and a *p*-value less than 0.05 was considered statistically significant. Continuous variables were expressed as means ± standard deviation and were compared using the unpaired *t*-tests. Continuous variables with non-Gaussian distribution were expressed with medians and interquartile range. Categorical variables were expressed as percentages and were compared with the χ^2^ test or Fisher’s exact test. Regarding hemodynamic improvement and end-organ perfusion, both POD-1 and POD-3 were compared to the pre-VAD baseline overall, and in survivors and non-survivors, respectively. A univariate analysis using multinomial logistic regression was employed to analyze the risk factors of mortality. Survival was calculated using the Kaplan–Meier method.

## 4. Results

**Figure 2:**
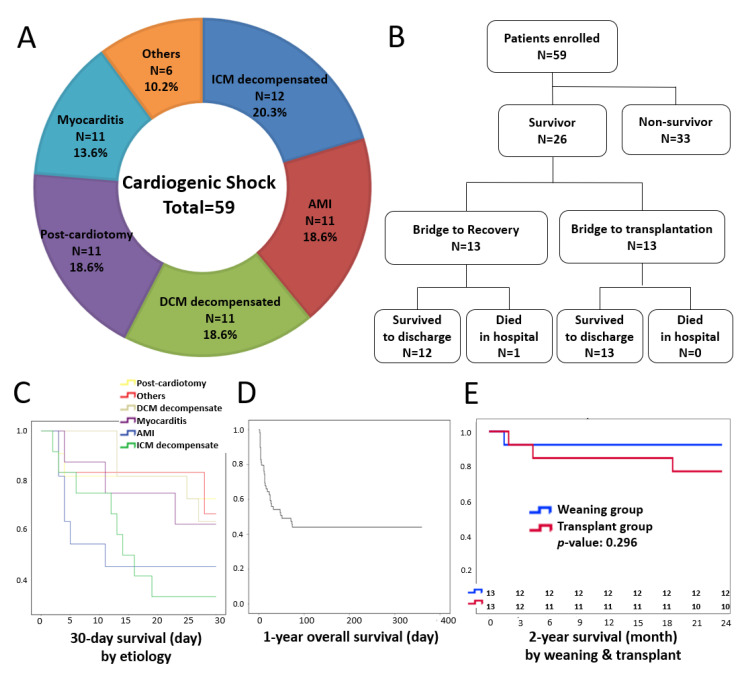
Patient destination and 30-day survival by etiology, 1-year overall survival, and 2-year survival in the weaning and transplant groups. (**A**) A pie chart depicting the etiology of cardiogenic shock. (**B**) A flow chart demonstrating the destinations of patients enrolled. (**C**) Survivals at 30 days were analyzed based on the etiology of cardiogenic shock. (**D**) Kaplan–Meier survival at one year was 40.6%. (**E**) The 2-year survival was 92.3% and 76.9% in the weaning and transplant groups, respectively.

A total of 59 consecutive patients with various etiologies of cardiogenic shock were enrolled between January 2015 and December 2019 (Figure 2A). Table 1 demonstrates the preoperative variables, including age, gender, body mass index (BMI), comorbidities, such as atrial fibrillation, diabetes, and hypertension, and previous percutaneous intra-aortic balloon pump (IABP). The mean CPR duration was 86.2 ± 22.1 min (range 60–155 min). Twenty (33.9%) patients had IABP support before ECLS. After ECLS implementation, 21 patients had ROSC, 18 had PEA/Asystole, and 20 had sustained VT/Vf. The mean left ventricle ejection fraction (LVEF) was 16.9 ± 6.56%. The median ECLS-to-VAD interval was 26 h (interquartile range 43 h). Only one patient required cardiopulmonary bypass (CPB) to remove a huge thrombus in the left atrium. In our series, the mean operating room duration was 232.7 ± 93.8 min. Oxygenators were spliced into the CentriMag system in six RVAD and fifty-three LVAD, respectively. The eventual mean LVAD output was 4.28 ± 0.84 L/min and the index to body surface area was 2.30 ± 0.50 L/min/m^2^. After t-BiVAD implementation, 10 patients were detected with pulsatile (pulse pressure), 33 had PEA/Asystole, and 16 had sustained VT/Vf. The mean LVEF was 11.3 ± 5.77%. The median VAD duration was 343 h (interquartile range 1004 h).

**Figure 3:**
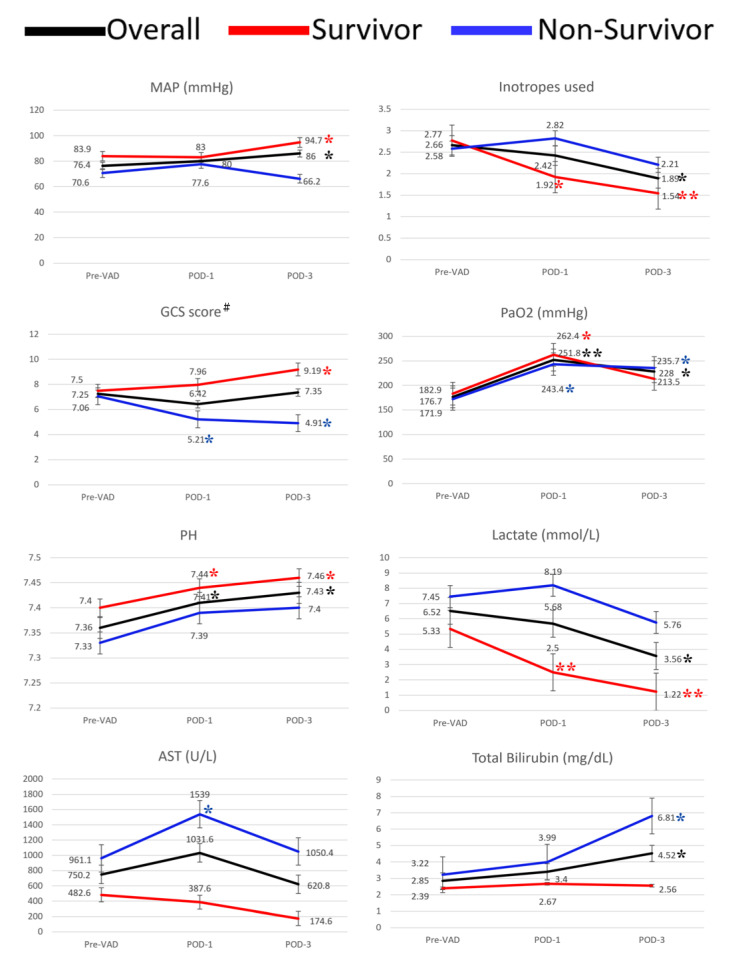
Improvement of hemodynamics and end-organ perfusion between the survivor and non-survivor groups. All POD-1 and POD-3 parameters were compared to pre-VAD (baseline) in the survivor subgroup, non-survivor subgroup, and overall group, respectively. Asterisks with different color represent the *p*-value in each corresponding group. * *p*-value < 0.05; ** *p*-value < 0.001. ^#^ All patients had endotracheal intubation with ventilator support. Thus, we were unable to assess verbal response. The GCS score only included eye opening and motor response. Verbal response was excluded in the scoring. VAD, ventricular assist device; POD, postoperative day; MAP, mean arterial pressure; GCS, Glasgow Coma Scale; PaO_2_, arterial oxygen tension; AST, aspartate aminotransferase.

Figure 3 shows the end-organ perfusion and hemodynamics before and after VAD implantation between the survivor and non-survivor groups. Overall, the mean arterial pressure (MAP) was significantly improved three days after VAD, with less than two inotropes, which was much more significant in the survival group. The GCS returned back to normal values in the survival group, but was significantly lower after VAD in the non-survival group. The N-terminal pro-brain natriuretic peptide (pro-BNP) was significantly reduced in both groups at POD-3. We did not observe any improvement in liver enzyme or renal laboratory parameters, as well as inflammation markers. The serum lactate was significantly decreased in the survival group and the arterial oxygen tension (PaO_2_) was significantly improved in both groups. The CentriMag-related major complications and causes of mortality are presented in Table 2 and Table 3. Major complications included critical bleeding (*n* = 22), systemic embolization (*n* = 5), infection (*n* = 4), and acute kidney injury (*n* = 41) (Table 2). There were 33 mortality cases, including 10 with central failure, 12 with multiple organ failure, and 11 with bacteremia and sepsis. Multinomial logistic regression predicted the related risk factors, including GCS, more than two inotropes, lactate ≥ 8 mmol/L at POD-1, and ventricular arrhythmia and total bilirubin ≥ 6 mg/dL at POD-3 (Table 3). Thirteen patients were bridged to recovery and thirteen were bridged to transplantation. One bridge-to-recovery case died in hospital due to persistent heart failure. There was no hospital mortality after transplantation. The overall 30-day survival and survival to discharge were 44.0% and 42.3%, respectively (Figure 2B,C), and the overall one-year survival was 40.6%. (Figure 2D). The 2-year survival was 92.3% and 76.9% in the weaning and transplant group, respectively (Figure 2E).

## 5. Discussion

The continuous-flow CentriMag pump (Levitronix LLC, Waltham, MA, USA) has a mag-levitated impeller design that has been reported to minimize friction and destruction of blood cells, preventing hemolysis and thrombus formation. A maximal blood flow of up to 9.9 L/min provides sufficient cardiac output to almost all Asian body sizes [8]. In Taiwan, this is the most commonly used temporary VAD device for end-stage heart failure, especially for bridge to decision in patients with cardiogenic shock. We used CentriMag with diverse configurations in different clinical scenarios (Figure 1B–E). All patients enrolled had suffered from CPR for more than one hour before ECLS, which always resulted in severe multiple organ dysfunction [9], as well as more than 90% mortality [2]. In emergency settings, the ECLS plays the role of salvage treatment with CPR to preserve end-organ perfusion (Figure 1B), especially preventing hypoxic brain damage [10,11]. At that point, GCS was only three and the neurologic deficits were always difficult to assess. Although CT scans demonstrated no brain tissue swelling before VAD, there were still 10 patients who developed central failure (Table 3). However, this also tells us that ECLS was very crucial in terms of ensuring sufficient cerebral perfusion despite any previous prolonged CPR. Therefore, these critical patients should not be abandoned too early, especially those whose CPR is witnessed and adequate. Consequently, VAD support should be initiated in cases of severe hypokinesia or akinesia of the LV. Indications include severe distension of LV with no opening of the aortic valve; swirl-smoke signs in the left side of the heart on echocardiography; differential hypoxia due to pulmonary edema; and of peripheral ECLS complications. In our treatment strategy, we would directly adopt BiVAD (Figure 1E) in patients having both CPR and ECLS, because they usually have both ventricle failure and congestive pulmonary edema. Further, most visceral organs also suffer from severe ischemic insult as well. In the first six cases with oxygenator spliced into RVAD, we found that oxygen might be extracted after pulmonary circulation. We assume this is due to the lower arterial saturation than the blood provided by the RVAD with oxygenator. However, this might be expected if the RVAD is not fully emptying the RA so that the oxygenated blood is mixed with deoxygenated blood that is pumped forward by the RVAD. Therefore, we spliced the oxygenator into LVAD in most cases enrolled (*n* = 53), which would help ischemic organs to obtain optimal oxygenation but also compensate the oxygen debt during CPR as soon as possible. Of course, with the RV function preserved, isolated LVAD is adequate for LV dysfunction only (Figure 1C). For bilateral ventricular failure without CPR or congestive pulmonary edema, we would adopt a C-configuration without oxygenator splicing (Figure 1D). This configuration is most commonly applied in patients with postcardiotomy syndrome, which could temporarily provide the cardiac output and allow waiting for the stunned myocardium to recover. However, if CPR or ECLS have already been applied, we would directly adopt a D-configuration instead of a C-configuration because patients in this group suffered from pulmonary congestion more or less. Once adequate oxygenation is reached after pulmonary edema resolution, the oxygenator could be easily removed at the bedside in the ICU.

Takeda et al. reported on a minimally invasive ventricular assist system with oxygenator that could spare the sternum, minimize bleeding, avoid severe adhesion, and convenience the subsequent durable VAD implantation or transplantation [3,12]. We have also experienced this sternum-sparing mini-invasive procedure, but we have faced some clinical dilemmas when it comes to Asian populations. First, the peripheral arteries cannulated with VAD, usually the subclavian and femoral arteries, are relatively small in Asian populations [13]. Percutaneous VAD, such as Impella, is not popular in our institution because the small-caliber peripheral arteries usually result in high incidence of acute ischemic limbs [14,15]. The configuration proposed from Dr. Takeda is always limiting perfusing flow, and high pressure always results in anastomosis bleeding. Of course, both subclavian and femoral arteries could be cannulated together simultaneously, but the groin is susceptible to infection [16], whereas mobilization is an additional issue to consider. Second, a single pump was used by Dr. Takeda for decompressing both ventricles in parallel; however, this approach could only perfuse systemic visceral organs rather than pulmonary vasculature. Insufficient pulmonary perfusion should be a concern if the RV is in akinesia. Third, the organ donation rate in Taiwan, as well as in the entire Asia–Pacific region, is significantly lower compared to Western countries [17,18]. As a result, the average waiting period for heart donation in our institution is between 2 and 3 months for patients with MCS and 1–3 years for patients without MCS. Of course, intracorporeal VAD is an additional choice for bridge to transplantation or for destination therapy. However, it is not reimbursed by Taiwan Health Insurance; thus, temporary VAD is the only feasible choice to bridge these patients either to transplantation or recovery. Although CentriMag has been approved for use for up to four weeks [19], several research studies have used CentriMag for more than 30 days [20,21,22]. In our study, the mean duration was 41.0 ± 37.4 days (range 8–115 days) in the bridge-to-recovery group, and 44.5 ± 45.9 days (range 2–171 days) in the bridge-to-transplantation group. As to the extended running of CentriMag, thromboembolic events raise another issue of concern. Frankly speaking, we did have some disastrous experiences with systemic embolization (Table 2), especially when the pump flow was less than 2.5 L/min. Thus, we applied systemic heparinization to target ACT 160–180 s as long as the mediasternal bleeding was less than 50 mL/hour 12 h after VAD implantation. Fourth, in our configurations (Figure 1D,E), we can arbitrarily splice and remove the oxygenator according to different clinical scenarios. Usually, the oxygenator could be removed after 3–5 days from pulmonary edema resolution. However, we do prefer to remove the oxygenator as soon as possible, because it might consume the coagulation factor and platelet, and induce coagulopathy [23,24]. In our experience, coagulopathy and bleeding tendency usually subsided immediately after removing the oxygenator. Takeda et al. provided an alternative mini-invasive procedure with a single pump and oxygenator for bi-ventricle support. We believe that this configuration is only indicated for extremely short-term cardiac support, which is also termed bridge to decision [25], and that these patients should be transferred to early emergent heart transplantation if possible. Otherwise, they should be transferred to durable LVAD, BiVAD, or even total artificial heart if transplantation is not feasible. In Taiwan, and Asia in general, the above-mentioned treatment is not as feasible as in developed Western countries. Thus, we designed a treatment algorithm for acute cardiogenic shock and decompensated chronic heart failure (Figure 1A). We assume that this is more practical in most Asian countries, where the organ donation rate is always low, as well as long-term VAD and total artificial heart are seldom available.

## 6. Limitations

First, this was a single-center study. The etiology of cardiogenic shock is too versatile and it is limited in performing intragroup and intergroup comparisons. Further, the results are based on our clinical practice policy, which may not be applicable to other heart centers. Second, the retrospective analysis utilized a historical chart review, and many unmeasured confounders might cause residual bias. However, it is difficult to design prospective randomized-controlled studies in this critical scenario. Third, a relatively small sample size and short-term follow-up may have also affected the validity of our findings. More cases and a longer-term follow-up period should be employed.

## 7. Conclusions

In light of the extremely poor prognosis of patients with prolonged CPR, combined ECLS and t-BiVAD could be a safe and effective treatment to rescue patients with cardiogenic shock and sequelae end-organ malperfusion. In our cohort, survival to weaning-off, to discharge, and 1-year survival were 44.0%, 42.3%, and 40.6%, respectively. We, furthermore, demonstrated versatile configurations and extended running, which allows for a sufficient time to bridge patients to recovery and heart transplantation.

## Figures and Tables

**Table 1:** Patient demographics.

	Mean ± SD/Median/Number	Range/Percentage/ Interquartile Range
**Comorbidity**		
Age (years)	51.7 ± 12.6	27–75
Female	11	18.6%
BMI (Kg/m^2^)	26.7 ± 5.9	15.5–45.3
BSA (L/min/m^2^)	1.88 ± 0.26	1.41–2.63
Atrial fibrillation	6	10.2%
Diabetes	19	32.2%
Hypertension	18	30.5%
Hyperlipidemia	27	45.8%
Valvular disease	30	50.8%
Coronary artery disease	34	57.6%
**Pre-ECLS**		
CPR duration (min)	86.2 ± 22.1	60–155
IABP	20	33.9%
**Post-ECLS, Pre-VAD**		
ROSC	21	35.6%
PEA/Asystole	18	30.5%
VT/Vf	20	33.9%
LVEF (%)	16.9 ± 6.56	5–30
ECLS-to-VAD interval (h) ^#^	26	43
**VAD operative demographics**		
Operating room duration (min)	232.7 ± 93.8	150–480
Oxygenator location		
Spliced into RVAD	6	
Spliced into LVAD	53	
LVAD output (L/min)	4.28 ± 0.84	1.93–6.05
LVAD output/BSA index (L/min/m^2^)	2.30 ± 0.50	1.13–3.56
**Post-VAD**		
Pulsatile blood pressure *	10	16.9%
PEA/Asystole	33	56.0%
VT/Vf	16	27.1%
LVEF (%)	11.3 ± 5.77	0–23
VAD duration (h) ^#^	343	1004

BMI, body mass index; BSA, body surface area; ECLS, extracorporeal life support; CPR, cardiopulmonary resuscitation; IABP, intra-aortic balloon pump; VAD, ventricular assist device; ROSC, return of spontaneous circulation; PEA, pulseless electrical activity; VT, ventricular tachycardia; Vf, ventricular fibrillation; LVEF, left ventricular ejection fraction; RVAD, right ventricular assist device; LVAD, left ventricular assist device; BSA, body surface area. ^#^ Continuous variables with non-Gaussian distribution were expressed as medians and interquartile range. * ROSC could not be distinguished due to continuous LVAD unloading to the left ventricle.

**Table 2:** Major complications between survivors and non-survivors.

Complications			
	Overall (%) (*n* = 59)	Number in Survivors (%) (*n* = 26)	Number in Non-Survivors (%) (*n* = 33)
**Critical Bleeding**	**22 (37.2)**	**9 (34.6)**	**13 (39.3)**
ICH	4 (6.7)	1 (3.8)	3 (9.0)
Pulmonary hemorrhage	2 (3.3)	0	2 (6.0)
Re-sternotomy for hemostasis ^#^	16 (27.1)	8 (30.7)	8 (24.2)
			
**Systemic Embolization**	**5 (8.4)**	**2 (7.6)**	**3 (9.0)**
Ischemic stroke	2 (3.3)	1 (3.8)	1 (3.0)
Ischemic bowel	2 (3.3)	0	2 (6.0)
Ischemic limb	1 (1.6)	1 (3.8)	0
			
**Infection**	**4 (6.7)**	**1 (3.8)**	**3 (9.0)**
Sepsis with DIC	2 (3.3)	0	2 (6.0)
Infective endocarditis	1 (1.6)	0	1 (3.0)
Deep sternal wound infection	1 (1.6)	1 (3.8)	0
			
**Acute Kidney Injury**	**41 (69.4)**	**17 (65.3)**	**24 (72.7)**
Temporary hemodialysis	37 (62.7)	13 (50)	24 (72.7)
Permanent hemodialysis	4 (6.7)	4 (15.3)	-

ICH, intra-cranial hemorrhage; DIC, Disseminated Intravascular Coagulation. ^#^ In the myocarditis group, four patients had re-sternotomy for bleeding, two of whom also underwent a second re-sternotomy.

**Table 3:** Causes and predicted risk factors of mortality.

**Cause of Mortality**		
	**Overall (*n* = 59)**	**Percentage (%)**
**Central failure** *	**10**	**16.9%**
Hypoxic encephalopathy	6	10.1%
Ischemic stroke	2	3.3%
ICH	2	3.3%
**Multiple organ failure**	**12**	**20.3%**
**Bacteremia with sepsis**	**11**	**18.6%**
**Total**	**33**	**55.9%**
**Multinomial Logistic Regression**		
**Risk Factors of Mortality**	**Odds Ratio**	**95% CI of the Difference**
**POD-1 (*n* = 59)**		
GCS ^#^ (Motor + Eye) ≤ 5	3.15	1.14–8.67
Two or more inotropes	6.70	0.83–54.1
Lactate ≥ 8 (mmol/L)	7.61	2.05–28.3
**POD-3 (*n* = 54)**		
Ventricular rhythm/asystole	5.90	1.83–18.98
Total bilirubin ≥ 6 (mg/dL)	8.12	1.69–39.0

ICH, intra-cranial hemorrhage; POD, postoperative day; GCS, Glasgow Coma Scale. * Central failure was diagnosed via repeat brain CT scan, as well as defined by the complete clinical neurologic examination, including documentation of coma, the absence of brain-stem reflexes, and apnea. ^#^ All patients had endotracheal intubation with ventilator support. Thus, we were unable to assess verbal response. The GCS score only included eye opening and motor response. Verbal response was excluded in the scoring.
